# Comprehensive analysis of autophagy related long non-coding RNAs in prognosis, immunity, and treatment of muscular invasive bladder cancer

**DOI:** 10.1038/s41598-022-13952-1

**Published:** 2022-07-04

**Authors:** Wei Tan, Ye Yuan, Hao Huang, Junhao Ma, Yadong Li, Yuanqing Gou, Hao Wu, Zili Hu

**Affiliations:** Department of Urology Surgery, The Second Affiliated Hospital of Chongqing Medical University, Chongqing, China

**Keywords:** Bladder cancer, Bladder, Prognostic markers, Data mining

## Abstract

To predict disease outcome in muscle-invasive bladder cancer (MIBC), we constructed a prognostic autophagy-related (PAR) lncRNA signature. Comprehensive bioinformatics analyses were performed using data from TCGA and GTEx databases. Univariate Cox, and least absolute shrinkage and selection operator regression analyses were also performed, based on differentially expressed genes, to identify PAR-related lncRNAs to establish the signature. Furthermore, the Kaplan–Meier OS curve and receiver operating characteristic curve analyses were performed and a nomogram was constructed, all of which together confirmed the strong predictive ability of the constructed signature. Patients with MIBC were then divided into high- and low-risk groups. Gene enrichment and immune infiltration analyses revealed the potential mechanisms in MIBC. We also further evaluated the signature of molecules related to immune checkpoints and the sensitivity toward chemotherapeutic agents and antitumor-targeted drugs to find better treatment prescriptions. We identified a number of PAR-related lncRNA signatures, including HCP5, AC024060.1, NEAT1, AC105942.1, XIST, MAFG-DT, and NR2F1-AS1, which could be valuable prognostic tools to develop more efficient, individualized drug therapies for MIBC patients.

## Introduction

Urothelial carcinoma, especially bladder cancer (BCa), is a common malignant tumor of the urinary system. Data from the Global Cancer Observatory (GCO, https://gco.iarc.fr/) shows that, 573,278 individuals were diagnosed with BCa and 212,536 individuals died of BCa in 2020, making BCa the fourth most common and tenth most fatal tumor worldwide^1^. It has also been reported that men are more susceptible to BCa (possibly due to smoking), making BCa the sixth most common carcinoma and ninth most fatal carcinoma in men. Most importantly, approximately 30% of BCa patients were diagnosed with muscular invasive bladder cancer (MIBC)^2^. Due to the high recurrence rate and poor prognosis of MIBC cases, patients usually experience great physical and mental stress and economic burden, which underscores the need to investigate new prognostic biomarkers in order to improve disease outcomes in patients through early detection and early treatment of high-risk patients.

Autophagy, as its name suggests, is a kind of cell “self-eating” and is prevalent as a self-protection mechanism of eukaryotic cells^3^. Autophagy leads to cell renewal and maintains homeostasis by degrading damaged organelles and macromolecules^4^. In cancer, autophagy can both promote and inhibit tumor progression, although the specific mechanism remains unclear.

Long non-coding RNAs (lncRNAs) are a type of ncRNA with transcripts > 200 nucleotides in length but with no protein-coding capacity^5^. lncRNAs participate in the occurrence and development of cancer through various biological processes, including the cell cycle, cell growth, cell death, drug resistance, and epigenetic regulation^5^.

Recently, the prevalence of autophagy-related lncRNAs, such as BLACAT1^6^, MALAT1^7^, XIST^8^, SNHGs^9^, HULC^10^, CASC2^11^, and GAS5^12^, was found to be associated with early diagnosis and prognosis of BCa, indicating that autophagy-related lncRNAs may be used as prognostic biomarkers and potential therapeutic targets. Therefore, we used autophagy-related lncRNAs to establish a prognostic autophagy-related (PAR) lncRNA signature for predicting disease outcome in BCa.

## Materials and methods

### Data acquisition

We acquired the RNA sequencing data of MIBC patients from the Cancer Genome Atlas (TCGA, https://cancergenome.nih.gov) and the Genotype-Tissue Expression Project (GTEx, https://www.genome.gov). Relevant clinical variables, such as age, sex, survival data, grade, and TNM stage, were also obtained from TCGA. We extracted 232 autophagy-related genes from the human autophagy database (HADb, http://autophagy.lu/clustering/index.html).

### Establishment and testing of the Risk Score Model

We randomly divided the MIBC patient samples from TCGA into training (n = 184) and testing (n = 180) groups (Table 1). The training cohort was used to establish lncRNA features and the testing cohort was used to verify the model. By applying differential expression analysis, univariate Cox regression analysis, least absolute shrinkage and selection operator (LASSO) regression analysis, Venn analysis, and multivariate Cox regression analysis, we identified seven PAR-lncRNAs which can be viewed as a signature to predict the disease outcomes of patients with MIBC. We established a lncRNA-mRNA co-expression network and Sankey diagram with the seven PAR-lncRNAs and 26 co-expressive mRNAs to visualize the potential relationship of the lncRNAs and mRNA.

**Table 1:** Clinical characteristics of patients in training and testing groups.

Characteristic	Train group		Test group	
	Number	Percent (%)	Number	Percent (%)
**n**	184	50.55	180	49.45
**Status**				
Alive	112	30.77	110	30.22
Dead	72	19.78	170	19.23
**Age, meidan**	68.67	18.87	67.77	18.62
< 70	94	25.82	95	26.10
≥ 70	90	24.73	85	23.35
**Gender**				
Male	141	38.74	129	35.44
Female	43	11.81	51	14.01
**Grade**				
High grade	173	47.53	170	46.70
Low grade	10	2.75	9	2.47
Unknown	1	0.27	1	0.27
**Stage**				
Stage II	52	14.29	48	13.19
Stage III	69	18.96	68	18.68
Stage IV	62	17.03	64	17.58
Unknown	1	0.27	0	0.00
**T stage**				
T2	62	17.03	55	15.11
T3	97	26.65	93	25.55
T4	25	6.87	42	11.54
**N stage**		0.00		0.00
N0	106	29.12	111	30.49
N1	19	5.22	24	6.59
N2	42	11.54	32	8.79
N3	2	0.55	4	1.10
Nx	15	4.12	9	2.47
**M stage**				
M0	90	24.73	84	23.08
M1	2	0.55	6	1.65
Mx	92	25.27	89	24.45
Unknown	0	0.00	1	0.27

Subsequently, we divided the training group into high- and low-risk groups using the median risk score as the cut-off point. Kaplan–Meier survival curve analysis was performed to show the difference in OS between the two groups. We also plotted the time-dependent receiver operating characteristic (ROC) curve and risk nomogram to evaluate the prediction accuracy of the model.

Furthermore, univariate and multivariate Cox analyses were performed to confirm that the seven PAR-lncRNAs signature was an independent prognostic factor for MIBC compared to other clinical characteristics—such as age, sex, clinical stage, TNM, and risk score. We analyzed the correlation between the risk scores and clinical parameters to investigate whether there was any difference in risk scores among different clinical parameter stratifications.

### Gene enrichment analysis

Gene ontology (GO) term enrichment analysis and Kyoto Encyclopedia of Genes and Genomes (KEGG) pathway analysis were performed to investigate potential signaling pathways and functions related to the seven lncRNAs included in the model^13–15^.

### Immune infiltration analysis and prediction of the sensitivity toward chemotherapeutic agents, antitumor targeted drugs, and immune checkpoint inhibitors

We used the R package “GSEABase” to investigate the difference in the expression and function of 16 infiltrating immune cells between the high- and low-risk groups. We also used the R package “pRRophetic” to analyze drug sensitivity in the high- and low-risk groups. In addition, we used the R package “ggpubr” to explore the sensitivity toward immune checkpoint inhibitors (ICIs) in the high- and low-risk groups.

### Statistical analysis

Cytoscape software (version 3.9.0) was used to construct an autophagy-related lncRNA co-expression network. R software (version x64 4.0.5) was used for statistical analysis, including Cox regression analysis, ROC curve analysis, gene enrichment analysis, and immune infiltration analysis.

## Results

### Data acquisition

The RNA sequencing and clinical data from the TCGA and GTEx databases included 375 MIBC patients and 28 para-cancerous tissues (19 from TCGA and 9 from GTEx). Differential expression analysis (|log2FC|> 2) revealed 175 differentially expressed lncRNAs in tumors compared with the corresponding expression in para-cancerous tissues.

We also extracted 232 autophagy-related genes from the human autophagy database (HADb, http://autophagy.lu/clustering/index.html), of which 112 were identified by the “limma” R package and Pearson correlation analysis.

### Risk Score Model

**Figure 1:**
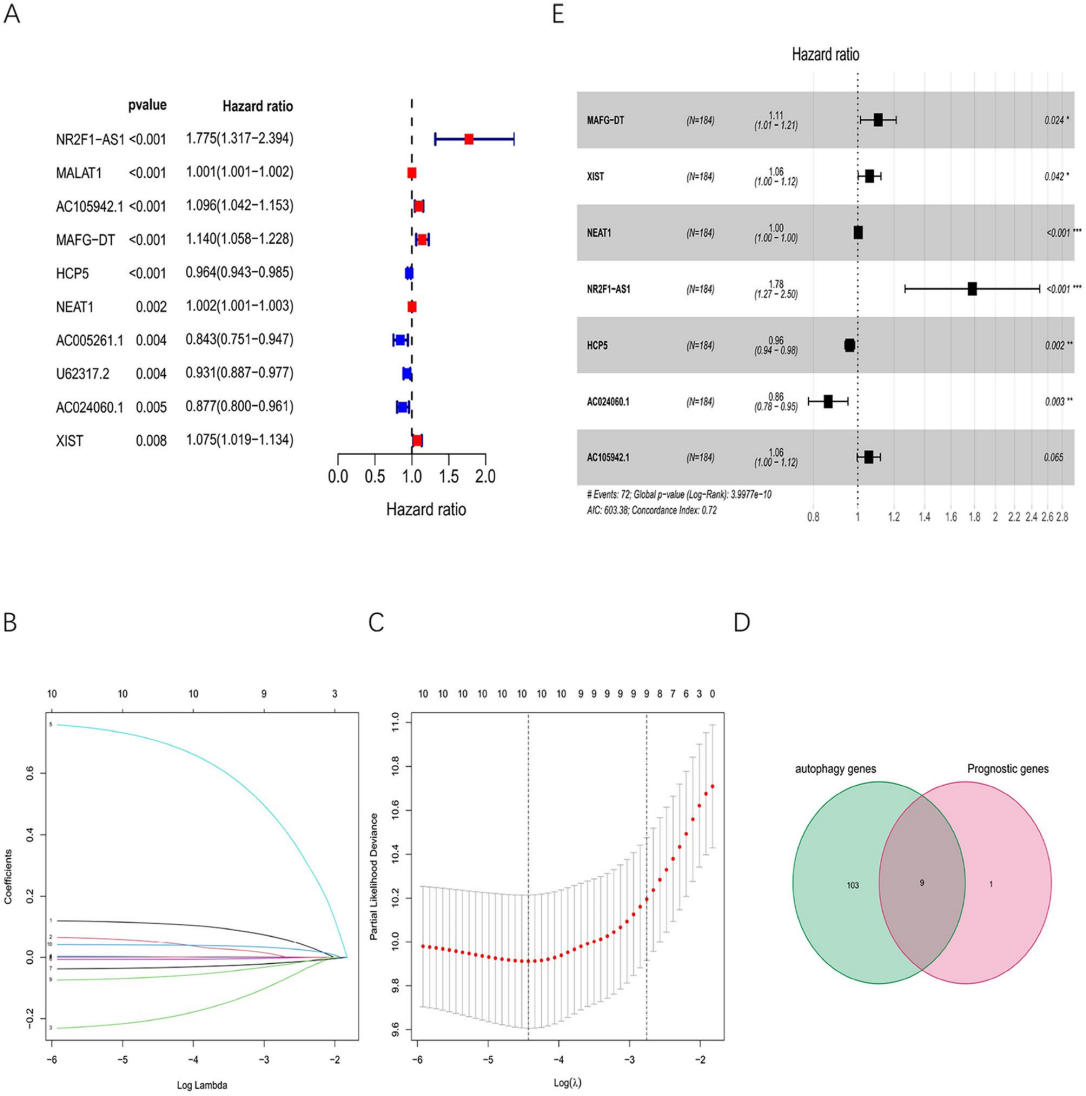
Selection of prognostic autophagy-related lncRNAs(PAR lncRNAs) with prognostic value. (**A**) Risk ratio forest plot shows that 10 autophagy-related lncRNAs, NR2F1-AS1, MALAT1, AC105942.1, MAFG-DT, HCP5, NEAT1, AC005261.1, U62317.2, AC024060.1, XIST, were significantly related to OS of MIBC patients from training group. (**B**) Adjusted parameters of LASSO regression model. (**C**) Figure for LASSO coefficient spectrum of prognostic lncRNAs. (**D**)The Venn analysis between DRGs and Prognostic genes. DRGs, differentially expressed genes. (**E**) Forest plot of multivariate Cox regression analysis shows the prognostic value of the 7 prognostic autophagy-related lncRNAs(PAR lncRNAs).

The MIBC samples from TCGA were randomly assigned to a training group (n = 184) and a testing group (n = 180). The training cohort was used to establish lncRNA features, and the testing cohort was used to verify the model. We then selected 10 prognosis-related lncRNAs from the training group for univariate Cox regression analysis (Fig. 1A). The least absolute shrinkage and selection operator (LASSO) regression analysis was then applied to further screen the 10 lncRNAs (Fig. 1B,C). Furthermore, we applied a Venn analysis to screen for nine possible PAR-lncRNAs (Fig. 1D). Multivariate Cox regression analysis showed that seven of the nine lncRNAs were suitable for the construction of a prognostic signature (Fig. 1E). Among the seven PAR-related lncRNAs included in the prognostic signature, HCP5 and AC024060.1 were considered protective factors (HR values less than 1), and NEAT1, AC105942.1, XIST, MAFG-DT, and NR2F1-AS1 were considered risk factors (HR values greater than 1).

In addition, we used the “survival” and “survminer” R packages to calculate the correlation coefficients. The risk score for the prognostic lncRNA model was calculated using the following formula:

$${\text{Risk}}\,{\text{score }} = \mathop \sum \limits_{{{\text{i}} = 1}}^{{\text{n}}} {\text{expression}}\,{\text{lncRNA}}\left( {\text{i}} \right)\, \times \,{\text{coefficient}}\,{\text{lncRNA}}\left( {\text{i}} \right)$$

The expression and coefficients of each lncRNA in the signature are shown in Supplementary Table 1.

### lncRNA-mRNA co-expression network

**Figure 2:**
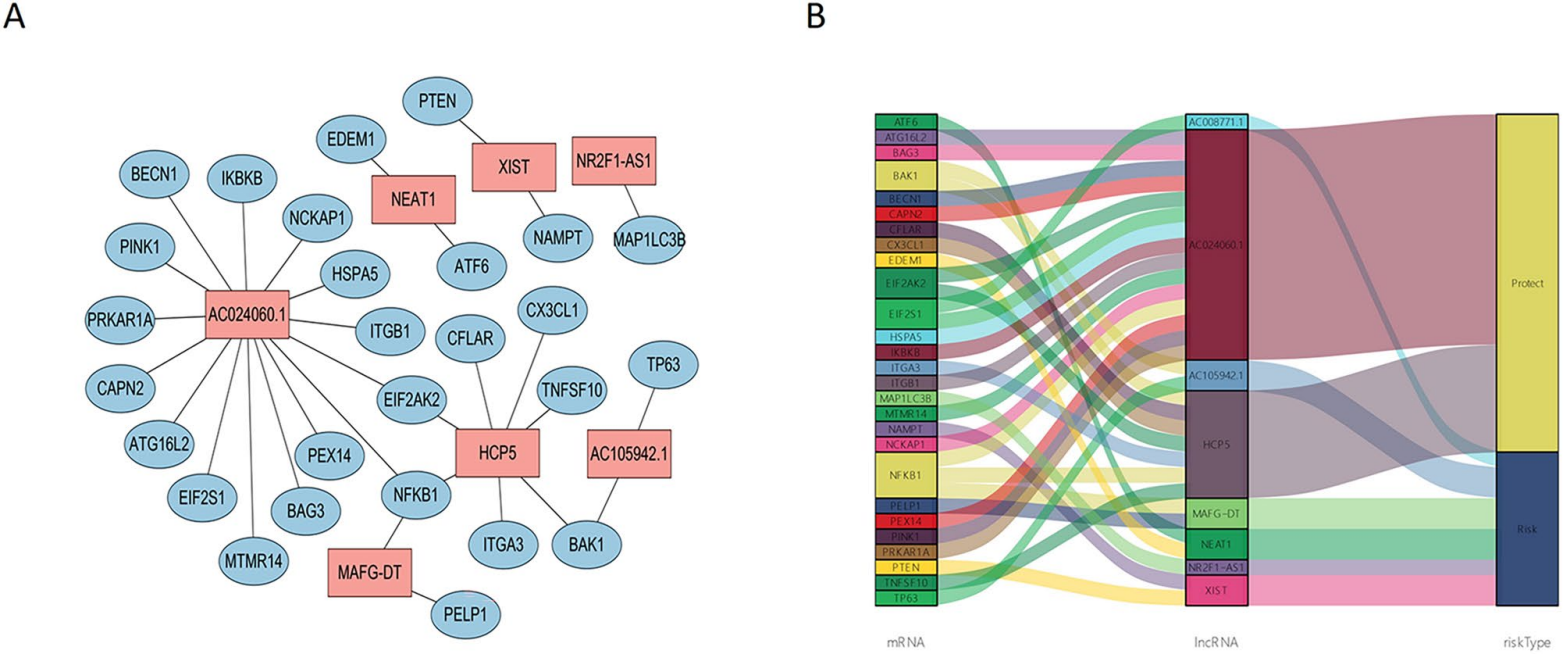
The establishment of lncRNA-mRNA co-expression network. (**A**) The PAR lncRNAs-mRNA co-expression network contains 7 PAR lncRNAs and 26 mRNAs with | coefficient value |> 0.3 and *P* value < 0.001; (**B**) the Sankey diagram shows the connection degree between the 7 PAR lncRNAs and 26 mRNAs (risk/protective). PAR lncRNAs, prognostic autophagy-related lncRNAs.

Once the seven PAR-lncRNAs were identified, we established a lncRNA-mRNA co-expression network to investigate the potential functions of the seven PAR-lncRNAs. The network was visualized using Cytoscape (Fig. 2A). The graph consisted of seven PAR-lncRNAs and 26 mRNAs (|coefficient value|> 0.3 and *P*-value < 0.001). Moreover, we drew a Sankey diagram to further display the risk/protective relationship between the seven PAR-lncRNAs and the 26 mRNAs (Fig. 2B).

### Testing of the Risk Score Model and independent prognostic analysis.

**Figure 3:**
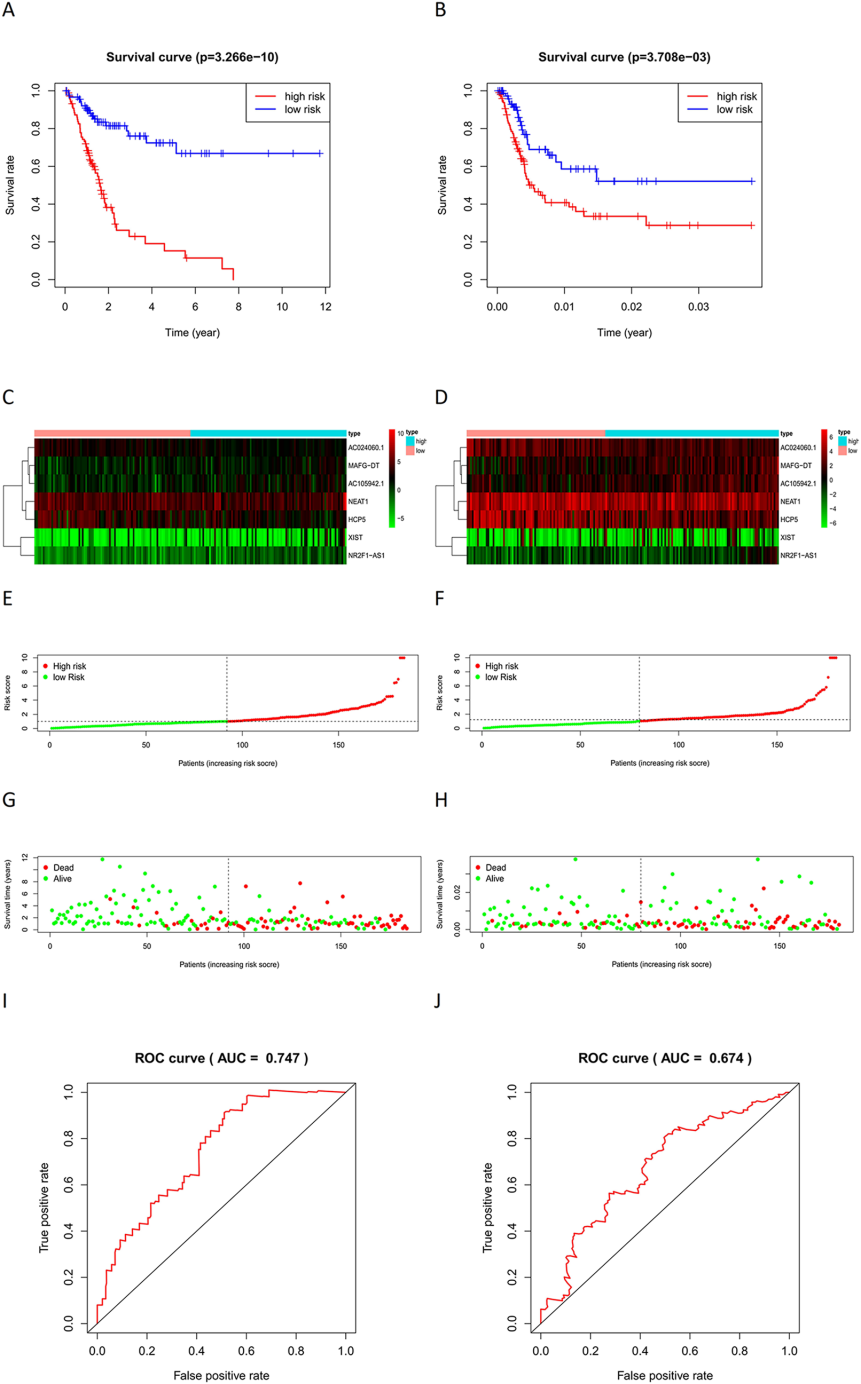
(**A**) The overall survival rate of the low-risk group was better than the high-risk group in the training group (*P* < 0.01); (**B**) the overall survival rate of the low-risk group was better than the high-risk group in the test group (*P* < 0.01); (**C**) the heatmap showed the differential expression of 7 PAR lncRNAs between the high- and low-risk groups in the training group; (**D**) the heatmap showed the differential expression of 7 PAR lncRNAs between the high- and low-risk groups in the test group; (**E**) the distribution of the risk score for every MIBC patient in the training group; (**F**) the distribution of the risk score for every MIBC patient in the test group; (**G**) the survival status of every MIBC patient with different risk scores in the training group; (**H**) the survival status of every MIBC patient with different risk scores in the test group; (**I**) the AUC of 1-year OS was 0.747 in the training group; (**J**) the AUC of 1-year OS was 0.674 in the test group; PAR lncRNAs, prognostic autophagy-related lncRNAs; MIBC, muscle-invasive bladder cancer; AUC, Area Under Curve; OS, Overall Survival.

**Figure 4:**
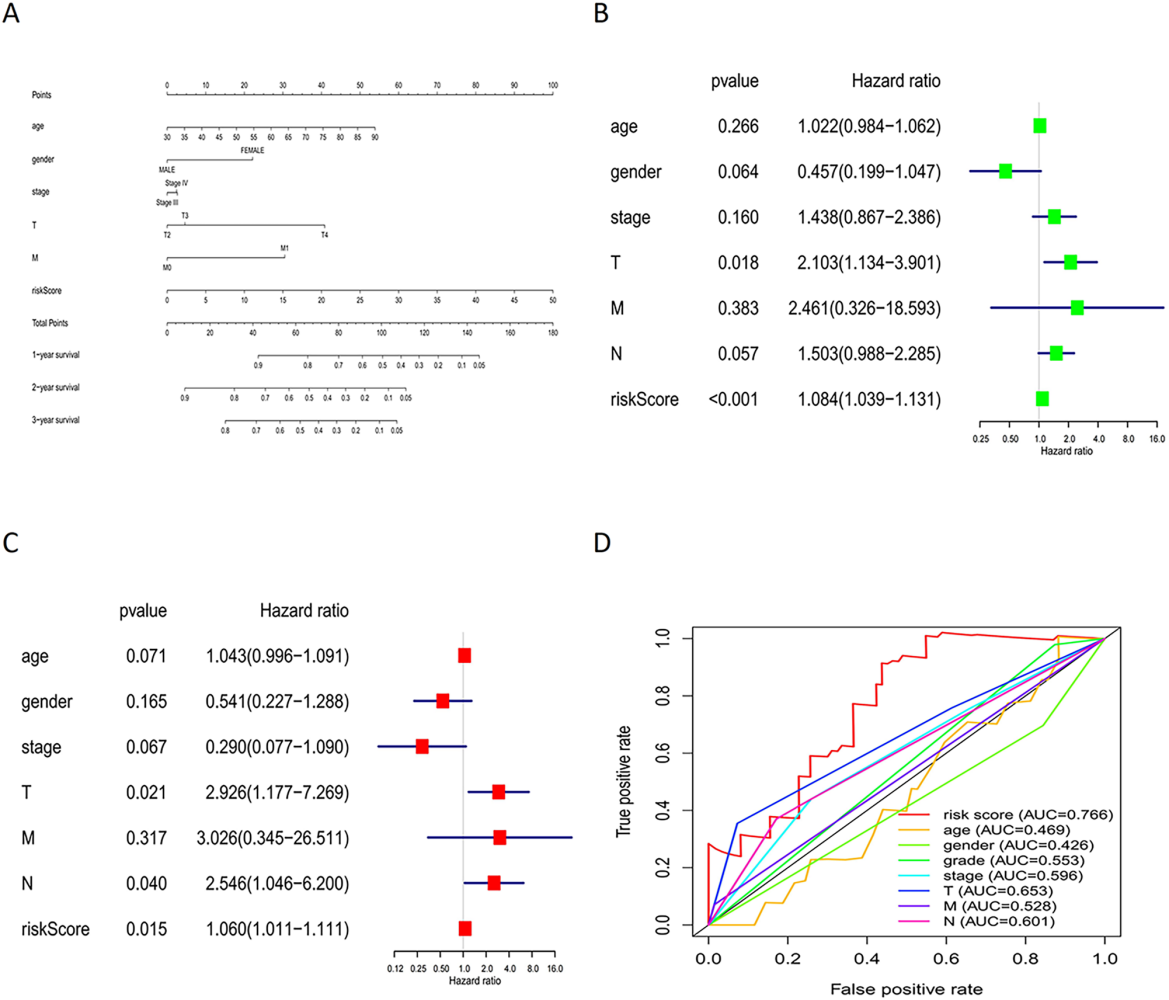
(**A**) Nomogram for predicting the 1- 3- and 5-year survival rates of MIBC patients; (**B**) Univariate Cox analysis reveals that clinical characteristics, like age, gender, clinical stage, TNM and risk score, were related to OS; (**C**) Multivariate Cox analysis of these clinical characteristics shows that the 7-PAR lncRNAs signature is an independent prognostic factor for MIBC; (**D**) The 7-PAR lncRNAs signature is an excellent predictive indicator of prognosis than any other clinical characteristic, with an area under the curve (AUC) of 0.766.

Patients in the training group were divided into high- and low-risk groups using the median risk score as the cut-off point. The results of the Kaplan–Meier survival curve analysis showed that the OS in the high-risk group was much lower than that in the low-risk group (Fig. 3A,B). For the training group, the expression heatmap of these seven PAR-related lncRNAs, the risk score of the signature in the low- and high-risk groups, and the survival time of MIBC patients are displayed in Fig. 3C,E,G. The same assessments were applied to the test group (Fig. 3D,F,H). We also drew a time-dependent ROC curve for the patients in the two groups (Fig. 3I,J). The area under the curve (AUC) values were used to evaluate the prediction accuracy of the model. In addition, a risk nomogram was established to further determine the predictive ability of the model including seven-PAR-lncRNA signature and univariate significant features, such as gender, age, T, M, and risk score (Fig. 4A). Univariate and multivariate Cox analyses showed that the seven-PAR-lncRNA signature may be a reliable predictor of OS in patients with MIBC. Univariate Cox analysis revealed that clinical characteristics—such as age, sex, clinical stage, TNM, and risk score—were related to OS (Fig. 4B). Multivariate Cox analysis of these clinical characteristics showed that the seven-PAR-lncRNA signature was an independent prognostic factor for MIBC (*P* < 0.05; Fig. 4C). The ROC curve showed that the seven-PAR-lncRNA signature is an excellent predictive indicator of prognosis compared to any other clinical characteristics, with an AUC value of 0.766 (Fig. 4D).

### Gene enrichment analysis

**Figure 5:**
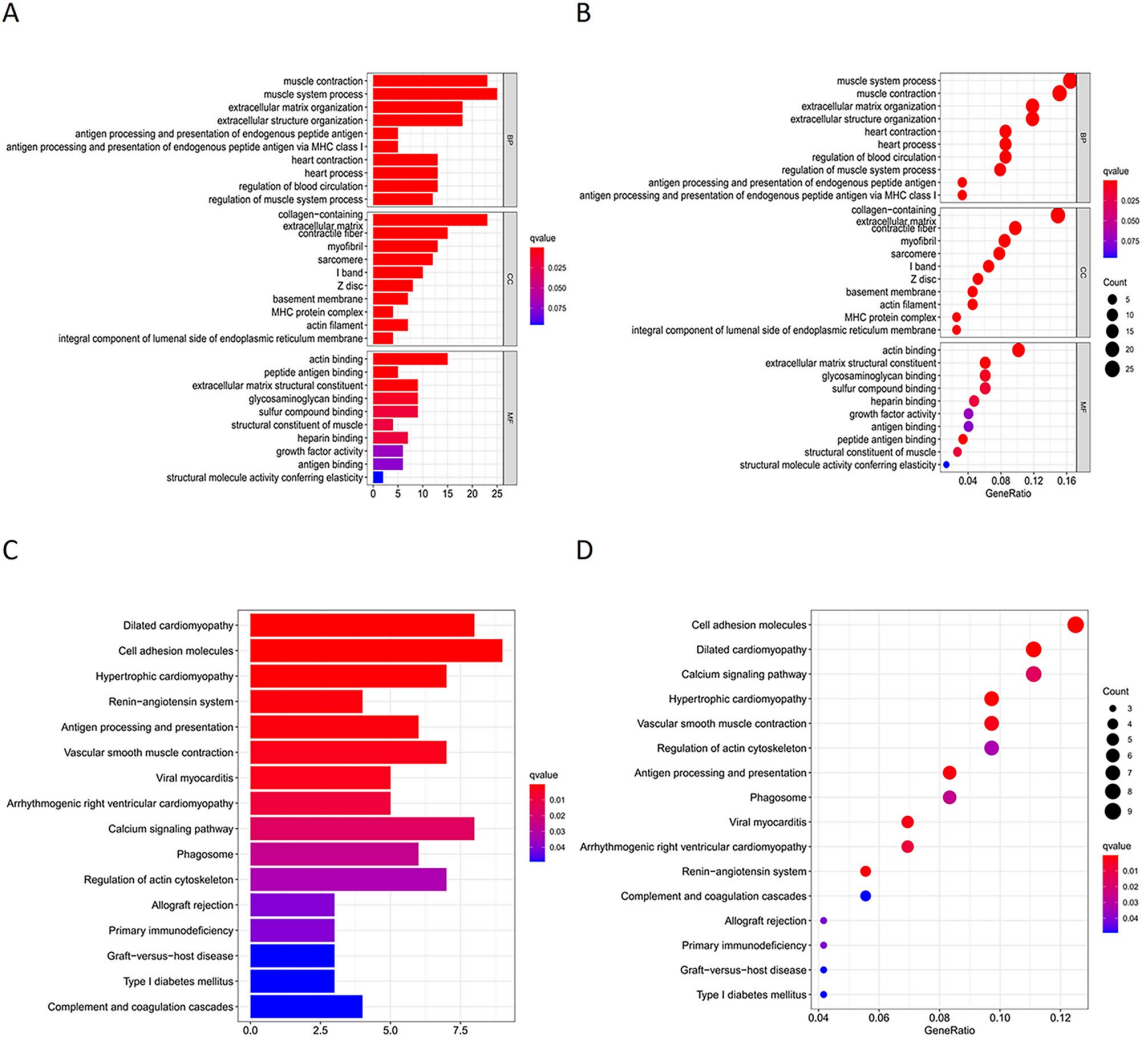
Gene enrichment analysis (**A**,**B**) Gene Ontology (GO) analysis results show the enriched biological processes, cell components and molecular functions associated with the mRNAs that co-express with the 7 prognostic autophagy-related lncRNAs. (**C**,**D**) Kyoto Encyclopedia of Genes and Genomes (KEGG) pathway analysis results shows the enriched signaling pathways associated with the mRNAs that co-express with the 7 prognostic autophagy-related lncRNAs.

To investigate the potential signaling pathways and functions related to the seven genes included in the model, we performed GO enrichment analysis (Fig. 5A,B) and KEGG pathway analysis (Fig. 5C,D). The GO enrichment analysis consists of three terms: biological process (BP), cellular component (CC), and molecular function (MF). In terms of BP, the seven ARGs with prognostic features were highly associated with muscle system processes, muscle contraction, extracellular matrix organization, and extracellular structure organization. In terms of CC, these genes were related to the collagen-containing extracellular matrix, contractile fiber, myofibril, and sarcomere. Furthermore, the major functions of these genes were revealed to be actin binding, extracellular matrix structural constituent, glycosaminoglycan binding, and sulfur compound binding. KEGG analysis showed that the seven PAR-related lncRNAs were mostly enriched for cell adhesion molecules, followed by dilated cardiomyopathy, hypertrophic cardiomyopathy, and vascular smooth muscle contraction.

### Clinical correlation analysis

**Figure 6:**
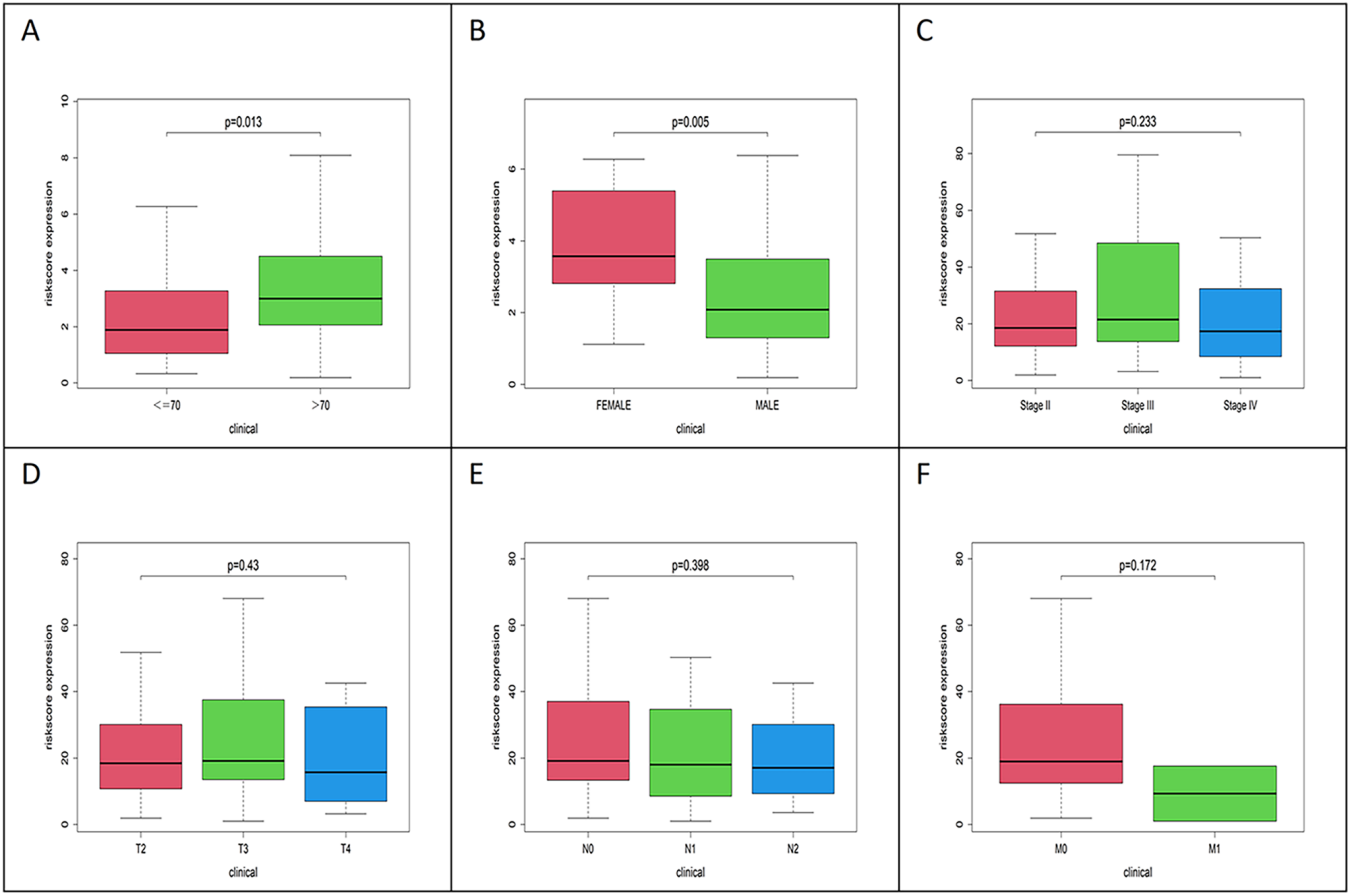
Correlation analyses of the prognostic autophagy-related lncRNAs signature with various clinicopathological characteristics of the MIBC patients. (**A**) patients aged > 70 have higher risk than those aged ≤ 70; (**B**) female have higher risk than male; (**C**) risk score has no statistical differences among stage II, III and IV; (**D**) risk score has no statistical differences among T2, T3 and T4; (**E**) risk score has no statistical differences among N0, N1, N2; (**F**) risk score has no statistical differences between M0 and M1.

We then analyzed the correlation between the risk scores and other clinical parameters in the case of the training group (having exact clinicopathological characteristics). The results showed that patients aged > 70 years were at a higher risk than those aged ≤ 70 years. A similar result was observed between females and males. The risk score had no statistical differences between stages T, N, and M (Fig. 6).

### Immune infiltration analysis and prediction of the sensitivity toward chemotherapeutic agents, antitumor targeted drugs, and immune checkpoint inhibitors

**Figure 7:**
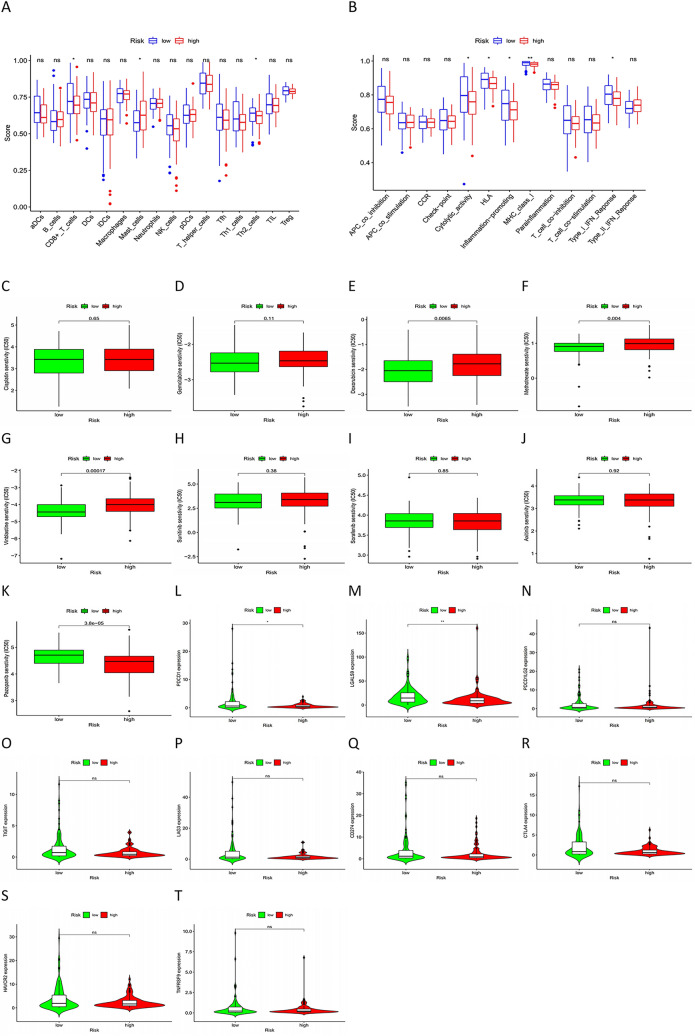
Immune infiltration analysis and prediction of the sensitivity of chemotherapeutic agents. (**A**) The expression of infiltrating immune cells has statistical difference in CD8+ T cells, Mast cells, Th2 cells between the high- and low-risk group; (**B**) The function of infiltrating immune cells has statistical difference in Cytolytic activity, HLA, Inflammation-promoting, MHC class I, Type I IFN Reponse between the high- and low-risk group; (**C**,**D**) In terms of drug sensitivity to cisplatin and gemcitabine, there is no statistical difference between high-risk group and low-risk group; (**E**–**G**) The sensitivity to Doxorubicin, Methotrexate, Vinblastine are much higher in high-risk group than in low-risk group. (**H**–**J**) The sensitivity to Sunitinib, Sorafenib, and Axitinib has no statistical difference between the high- and low- risk groups; (**K**) The sensitivity to Pazopanib is much higher in high-risk group than in low-risk group; (**L**) The expression level of PDCD1(PD-1) was remarkably higher in the low-risk group with *P* < 0.05; (**M**) The expression level of LGALS9 (GAL9)were remarkably higher in the low-risk group with *P* < 0.01; (**N**–**T**) The expression levels of PDCD1LG2 (PD-1LG2), TIGHT, LAG-3, CD274 (PD-L1), CTLA-4, HAVCR2 (TIM-3) and TNFRSF9 had no statistical difference between the high- and low- risk groups with the *P* > 0.05.

We used the R package “GSEABase” to investigate the difference in the expression and function of 16 infiltrating immune cells between the high- and low-risk groups. We found statistically significant differences in the expression of CD8+ T cells, mast cells, and Th2 cells (Fig. 7A). Furthermore, our findings also showed statistical differences in cytolytic activity, HLA, inflammation-promoting, MHC class I, and type I IFN Response (Fig. 7B).

To identify a more effective approach to treating MIBC, we analyzed the sensitivity of the high- and low-risk groups toward chemotherapeutic agents using the R package “pRRophetic”. From the boxplots, we found that there was no difference in sensitivity toward gemcitabine and cisplatin between the two groups (Fig. 7C,D), whereas higher sensitivity was observed for doxorubicin, methotrexate, and vinblastine in the high-risk group than in the low-risk group (Fig. 7E–G). This indicates that patients in the high-risk group may be more likely to gain better efficacy with the MAVC drug regimen (consisting of cisplatin, doxorubicin, methotrexate, and vinblastine) regimen rather than with the GC regimen (gemcitabine and cisplatin).

Antitumor drugs, such as sorafenib and sunitinib, have been shown to have an excellent effect on renal cell carcinoma. However, in BCa, the effect of these antitumor-targeted drugs, excluding erdafitinib, is still unclear. Accordingly, we investigated the sensitivity of these cells to common antitumor-targeted drugs. The data from these studies showed that there was no statistically significant difference between the high- and low-risk groups in terms of their sensitivity to sunitinib, sorafenib, and axitinib (Fig. 7H–J) and that the sensitivity to pazopanib was much higher in the high-risk than in the low-risk group (Fig. 7K).

We also compared the high- and low-risk groups for differences in the sensitivity toward immune checkpoint inhibitors (ICIs), such as pembrolizumab and atezolizumab, which have been reported to show excellent durable responses and disease control in patients with BCa^16,17^. We utilized the "limma" and "gpubr" packages to generate violin plots. As can be seen in Fig. 7M, the expression levels of PDCD1(PD-1) (*P* < 0.05, Fig. 7L) and LGALS9 (GAL9) (*P* < 0.01) were remarkably higher in the low-risk group. The expression levels of PDCD1LG2 (PD-1LG2), TIGHT, LAG-3, CD274 (PD-L1), CTLA-4, HAVCR2 (TIM-3), and TNFRSF9 were not significantly different between the high- and low-risk groups (*P* > 0.05, Fig. 7N–T).

Altogether, these results seem to indicate that there is significant potential for improving therapeutic regimens for MIBC.

## Discussion

BCa is one of the most common tumors of the urinary system, and data from the Global Cancer Observatory shows that the number of new BCa patients in 2020 is ranked fourth among that of all malignancies^1^. Among all patients diagnosed with BCa, approximately 30% have MIBC^2^, which has a poor prognosis. Although various therapeutic regimens ameliorate the effects of the disease, there is room for improvement in terms of categorizing patients for more individualized treatment and a better prognosis. That is why we carry on this study. By this signature, patients will be divided into high- and low-risk groups, and then they can get individualized treatment plans, considering the result of drug sensitivity, so that they can gain a better prognosis.

Recently, researchers have found that autophagy and lncRNAs play important roles in the occurrence and development of cancer^18–23^. In the present study, we established a seven-PAR-lncRNA signature, based on the data of MIBC patients from TCGA, to predict the prognosis of MIBC patients. We evaluated the prediction accuracy of the signature using Kaplan–Meier survival curve analysis, time-dependent ROC curve, risk nomogram, univariate and multivariate Cox analyses, and other analyses. In examining the drug sensitivity, we found that patients in the high-risk group may be more likely to benefit from the MAVC regimen than by the GC regimen, which suggests that MAVC should be given higher priority over GC when choosing a chemotherapy regimen for high-risk patients.

In our study, HCP5 and AC024060.1 were considered protective factors, with HR values less than 1. NEAT1, AC105942.1, XIST, MAFG-DT, and NR2F1-AS1 were considered as risk factors with HR values greater than 1. Therefore, we searched the published literature for these genes.

HCP5 can negatively regulate the expression of miR-29b-3p to promote BCa cell viability, migration, and invasion^24^. HCP5 is a part of an immune-related lncRNA signature, which is related to the prognosis of BCa^25^. AC024060.1 could be seen in the other two prognosis-related signatures of BCa; however, research on the specific mechanism is still lacking^26,27^.

NEAT1, located on chromosome 11, is a structural component of nuclear paraspeckles, which plays a significant oncogenic role in proliferation and cell migration^28^. It has been shown that the overexpression of NEAT1 is associated with poor prognosis of many cancers^29^. Shan et al. reported that NEAT1 promotes bladder progression through the NEAT1/miR-410/HMGB1 axis^30^. NEAT1 has also been shown to play an important role in the treatment of BCa. Zhao et al. found that the suppression of cell growth, invasion, and apoptosis of BCa cells under cisplatin chemotherapy could be enhanced by silencing NEAT1^31^. Similarly, by negatively regulating miR-214-3p expression, NEAT1 promotes doxorubicin resistance in BCa via the Wnt/β-catenin pathway^32^.

XIST, located in the X chromosome inactivation center of Xq13.2^33^, has been shown to play a wide range of roles in the occurrence and progression of therioma^34,35^. The results of previous studies have indicated that XIST can participate in the occurrence, progression, and metastasis of therioma via the XIST-TET1-p53 regulatory network^36^, miR-133a^37^, XIST/miR-200c^38^, miR-124 dependent androgen receptor regulation^39^, and the miR-139-5p mediated Wnt/β-catenin signaling pathway^40^.

In another two prognosis-related signatures of BCa, AC105942.1 was also revealed to be a part of the signatures, but the specific mechanism of action of AC105942.1 in cancer is still unclear^41,42^. A similar conclusion was also found for MAFG-DT^42–44^ and NR2F1-AS1^45,46^.

In recent years, the treatment of BCa has become increasingly diversified, including surgery, chemotherapy, immunotherapy, and targeted therapy. Here, we explored alternative treatments for MIBC that could be more effective than the existing ones.

Chemotherapy plays an important role in MIBC treatment. GC and MVAC are the most commonly used chemotherapeutic regimens. Through sensitivity tests of these drugs, we found that the high-risk MIBC patients had a much higher sensitivity toward MVAC than that toward GC, which indicates that patients in the high-risk group may be more likely to benefit from the MAVC regimen rather than the GC regimen. Similar tests were performed for antitumor-targeted drugs for urothelial carcinoma and immune checkpoint inhibitors. We hope that these results will help clinicians choose more effective, treatment options for individual patients.

However, our study has some limitations. First, external validation of the signature in large-scale multicenter cohorts is necessary before the signature is widely adopted, owing to the limitations of the data source and sample size of our study. In addition, although we determined that these seven lncRNAs play an important role in BCa, the precise mechanism of action remains unclear. Further functional experiments are required to elucidate the mechanisms of these genes. In summary, further studies are needed to elucidate the mechanisms and effects of MIBC.

## Conclusion

Utilizing MIBC patient data obtained from TCGA, we established a seven-PAR-lncRNA signature to predict the disease outcome of patients with MIBC. Furthermore, we explored alternative pharmaceutical treatments that could be individualized for MIBC patients based on disease risk. Our results indicate that patients in the high-risk group may benefit more from the MAVC drug regimen than the GC regimen, pazopanib may be more effective in high-risk MIBC patients, and drugs acting on PD-1 and LGALS9 gene loci may be more effective in low-risk patients.

## Supplementary Information

Supplementary Information.

## Data Availability

All the data in this study can be found in The Cancer Genome Atlas(TCGA, https: //cancergenome.nih.gov)and Genotype-Tissue Expression Project (GTEx, https://www.genome.gov).
